# Practice makes perfect: the neural substrates of tactile discrimination by Mah-Jong experts include the primary visual cortex

**DOI:** 10.1186/1471-2202-7-79

**Published:** 2006-12-05

**Authors:** Daisuke N Saito, Tomohisa Okada, Manabu Honda, Yoshiharu Yonekura, Norihiro Sadato

**Affiliations:** 1 Division of Cerebral Integration, National Institute for Physiological Sciences, Okazaki, Japan; 2 JST (Japan Science and Technology Corporation)/RISTEX (Research Institute of Science and Technology for Society), Kawaguchi, Japan; 3 Department of Cortical Function Disorders, National Institute of Neuroscience, National Center for Neurology and Psychiatry, Tokyo, Japan; 4 Biomedical Imaging Research Center, University of Fukui, Fukui, Japan; 5 Department of Functional Neuroimaging, Faculty of Medical Sciences, University of Fukui, Fukui, Japan

## Abstract

**Background:**

It has yet to be determined whether visual-tactile cross-modal plasticity due to visual deprivation, particularly in the primary visual cortex (V1), is solely due to visual deprivation or if it is a result of long-term tactile training. Here we conducted an fMRI study with normally-sighted participants who had undergone long-term training on the tactile shape discrimination of the two dimensional (2D) shapes on Mah-Jong tiles (Mah-Jong experts). Eight Mah-Jong experts and twelve healthy volunteers who were naïve to Mah-Jong performed a tactile shape matching task using Mah-Jong tiles with no visual input. Furthermore, seven out of eight experts performed a tactile shape matching task with unfamiliar 2D Braille characters.

**Results:**

When participants performed tactile discrimination of Mah-Jong tiles, the left lateral occipital cortex (LO) and V1 were activated in the well-trained subjects. In the naïve subjects, the LO was activated but V1 was not activated. Both the LO and V1 of the well-trained subjects were activated during Braille tactile discrimination tasks.

**Conclusion:**

The activation of V1 in subjects trained in tactile discrimination may represent altered cross-modal responses as a result of long-term training.

## Background

Visual-tactile cross-modal plasticity due to visual deprivation has been reported [1-5]. For visually-deprived persons, Braille is the most successful system for the transmission of written information. In Braille, the visual perception of printed characters is replaced by the tactile interpretation of raised dots. Braille reading is known to activate the visual cortex of the blind, indicating remarkable cortical plasticity. However, such findings typically come from experiments in subjects who have been blind from a very early age and who also have undergone Braille training from a young age. As a result, it is not clear whether the visual cortex activation is related to long-term Braille training or to visual deafferentation. A previous study has shown that non-Braille haptic processes activate the visual cortex of blind subjects who read Braille proficiently, which is consistent with the latter hypothesis [4]. Additionally, the occipital cortex of blind subjects without Braille training was activated during tactile discrimination tasks, whereas that of control sighted subjects was not [4]. Hence the activation of the association visual cortex of blind subjects while performing a tactile discrimination task may be due to sensory deafferentation, wherein a competitive imbalance favors the tactile over the visual modality. Recent studies indicate that both tactile and visual processing of objects are represented in the visual association cortex [6,7], indicating that visual and tactile processing may be competitively balanced in the association cortices where the inputs adjoin [8]. Therefore, visual deafferentation may cause less demand on bottom-up visual processing, which may in turn introduce an opportunity for the expansion of tactile representations in the visual association cortex.

These findings indicate that cross-modal plasticity may be induced not only by sensory deafferentation but also by learning. Recently, audio-visual cross-modal plasticity due to learning has been shown while pianists observe piano playing [9]. Well-trained pianists are able to identify pieces of music by watching the hands touch the piano keys; thus the visual information from observing the sequential finger movements was transformed into the auditory modality during "key-touch reading". Viewing the bimanual hand movements of a piano player making key presses actually activated the left planum temporale (PT) of well-trained subjects. The naïve and less well-trained groups did not show activation of the left PT during any of the tasks [9].

Anticipating a similar learning effect in visual-tactile cross-modal plasticity, we conducted fMRI studies in persons with normal sight who have had long-term tactile shape discrimination training on Mah-Jong tiles [10]. Mah-Jong is a Chinese game similar to card games, involving two-dimensional plastic tiles with various marks carved on one side. Some well-trained Mah-Jong players can identify the carved patterns by touch. Our hypothesis was that the subjects who are well-trained on the tactile discrimination of Mah-Jong patterns would show more prominent activation in the visual cortex when performing this task, including the multi-modal ventral association visual cortex, than the naïve subjects. Furthermore, as we expected the same effect even with unfamiliar materials, the Mah-Jong experts also underwent a tactile discrimination task using Braille characters, to which they were naïve.

## Results and discussion

The mean percentage of correct responses during Mah-Jong tile discrimination was 88.8 ± 6.9% (mean ± SD) in the expert group, which was significantly better than the control group (70.0 ± 19.3%, *P *= 0.01, two-sample t-test). The mean accuracy during Braille discrimination by the seven subjects in the expert group was 72.9 ± 7.6%, which was significantly worse than the Mah-Jong discrimination (87.9 ± 7.0%) by the same subjects (*P *= 0.003, paired t-test), and comparable with the accuracy scores of the control group during Mah-Jong discrimination (*P *= 0.68, two-sample t-test).

**Figure 1:**
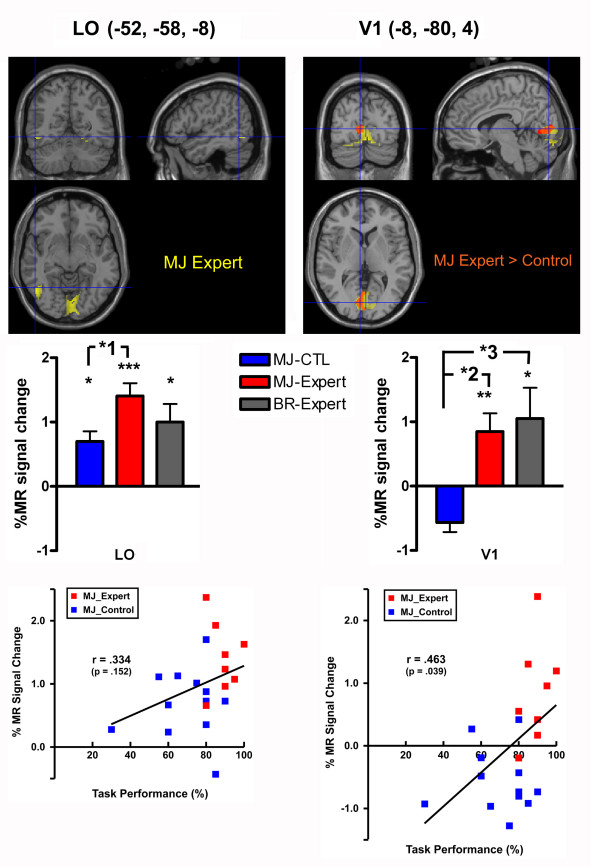
(Top row) Statistical parametric maps of the group analysis of the neural activity of the expert subjects during tactile Mah-Jong discrimination. Task-related increases in the MR signal (yellow) were superimposed on three orthogonal sections of T1-weighted high-resolution MRIs unrelated to the subjects of the present study. The fMRI data were normalized to standard stereotaxic space. The blue lines indicate the projections of each section that cross at (x = -52 mm, y = -58 mm, z = -8 mm in MNI coordinates) where the activation was maximal in the LO when the experts were completing the task. (Top right) The group difference between the experts and the non-expert control group during Mah-Jong tactilediscrimination (orange) within the areas activated by expert group(yellow) are shown in the same format. The V1 of the expert groupshowed more prominent activation than that of the control group(orange). The blue lines indicate the projections of each section that cross at (-8, -80, 4) where the group difference was maximal. (Middle row) Task-related activation in the left LO (-52, -58, -8) (middle left), and V1 (-8, -80, 4) (middle right). The percent MR signal change during Mah-Jong discrimination by the non-expert control group (blue bar) and the experts (red bar) were plotted. The percent MR signal change during Braille discrimination by seven out of the eight experts (shaded bar) is also presented. * <0.05, ** <0.01, *** <0.001, one sample *t*-test. The group difference was also significant (*P = 0.02; ** *P *= 0.001; *** *P *= 0.003, Mann Whitney U-test). (Bottom row) The % MR signal change during Mah-Jong tactile discrimination by the expert (red square) and control groups (blue square) in the LO (left) and V1 (right). In V1 the task-related activity and task performance was positively correlated.

**Figure 2:**
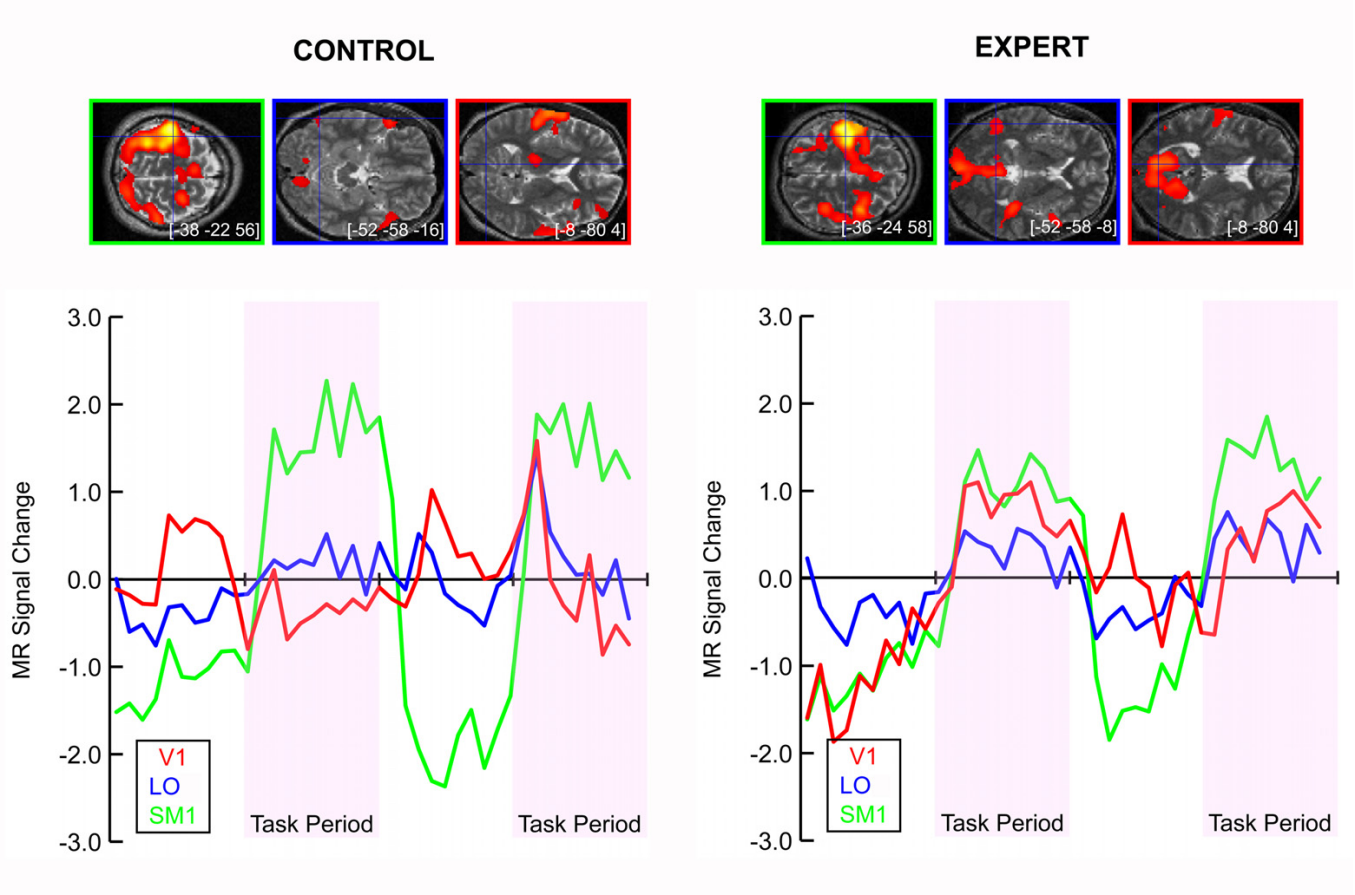
Individual analysis of the task-related activation in the left S1 (green), left LO (blue), and V1 (red). (Top) The foci with significant activation during Mah-Jong tactile discrimination by a non-expert (left) and an expert (right) were superimposed on the transaxial images of the T2-weighted high-resolution MRI of each subject. Blue lines indicate the projection of the yz and xz sections in standard stereotaxic space at the MNI coordinates indicated in each image. (Bottom) The time courses of the MR signal changes of each location (V1 in red, LO in blue, and SM1 in green) were plotted. The mean value of the time-course data of each plot was centered to zero.

As our region of interest was the occipital cortex, we searched for task-related activation in the occipital lobe. In the expert group there was significant occipital midline activation, possibly incorporating the primary visual cortex (V1) and the lateral occipital cortex (LO) on the left (*P *< 0.05, corrected for multiple comparisons, Table 1, Figure 1). Both groups showed significant activation (*P *= 0.013 for the Control group, *P *< 0.001 for the Expert group; one sample t-test, Figure 2) in the left LO (Montreal Neurological Institute (MNI) coordinates, x = -52 mm, y = -62 mm, z = -8 mm) [11]. Compared with the control group, the primary visual cortex (MNI coordinates, x = -8 mm, y = -80 mm, z = 4 mm, *P *= 0.001, Mann Whitney U-test) and the left LO (MNI coordinates, x = -52 mm, y = -62 mm, z = -8 mm, *P *= 0.02, Mann Whitney U-test) of the expert group were more prominently activated (Figure 1). There was no significant activation in the occipital cortex when we contrasted CTL > MJ experts.

**Figure 3:**
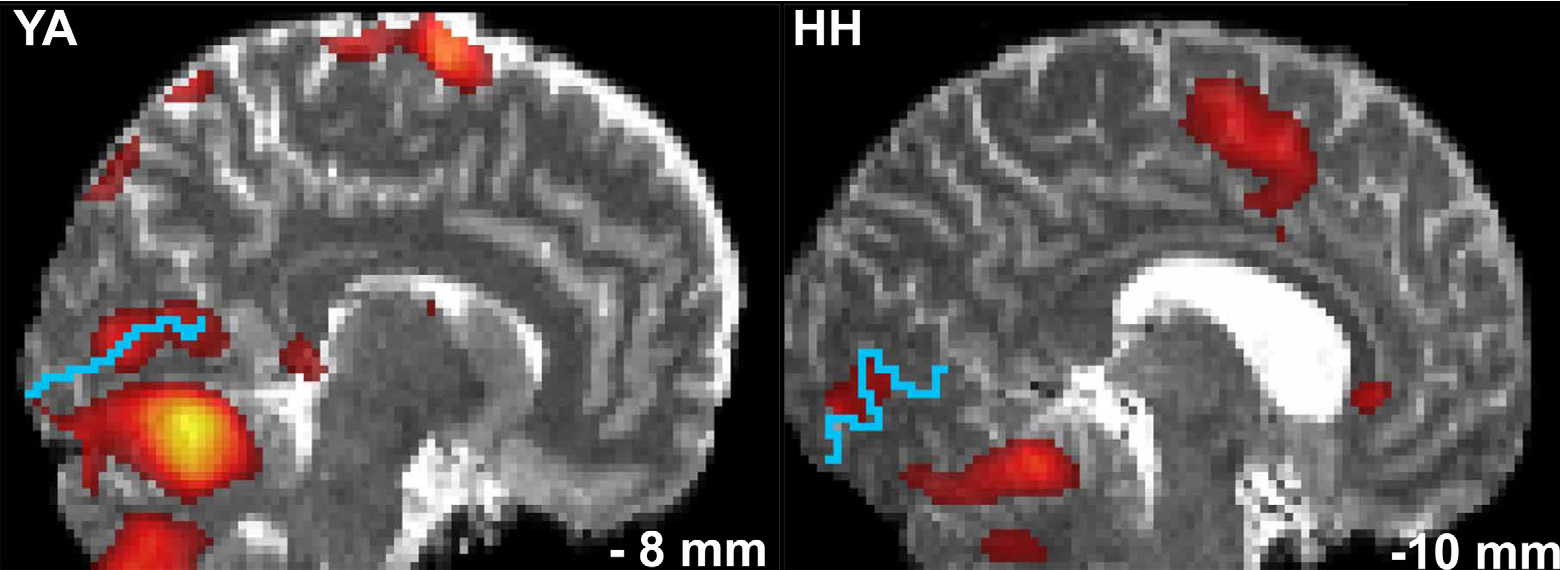
Individual analysis of the task-related activation in the V1. The foci with significant activation during Mah-Jong tactile discrimination by experts (YA, left; HH, right) were superimposed on the parasagittal sections (MNI coordinates of x = -8 mm for YA, and x = -10 mm for HH) of the T2-weighted high-resolution MRI of each subject. Blue lines indicate the calcarine sulcus.

The expert group also revealed significant activation in the left LO (*P *= 0.011, one-sample t-test) and in V1 (*P *= 0.035, one sample t-test) during the Braille task. The activation of V1 by the Braille task in the Expert group was significantly more prominent than that in the Control group by the Mah-Jong task (*P *= 0.003, Mann Whitney U-test, Figure 1). Although our main interest was in the occipital cortex, we also evaluated the parietal cortex. There was no significant group difference in the parietal cortex, particularly in the primary somatosensory area (S1) (MNI coordinates, x = -36 mm, y = -24 mm, z = 58 mm). The time course of the S1, V1, and LO activation of a representative subject from each group is presented in Figure 2. When we included the non-experts, performance on the Mah-Jong tactile discrimination task and the V1 activity were positively correlated (Figure 1). Typical individual data are shown in Figure 3, revealing that the Mah-Jong discrimination activated the regions adjacent to the calcarine salcus, and hence V1 (Figure 3). Furthermore, the inter-subject variation of calcarine sulcus with respect to the distance from the local maxim of interest, (-8, -80, 4), was small: The distance was 2.3 mm +/- 2.4 mm (N = 20) calculated with the y-z plane of the normalized individual anatomical MRI. This study showed that the left lateral occipital cortex was activated by the tactile shape discrimination task. Based on Talairach's coordinates [12] – converted from MNI coordinates using an established formula [13] – this area corresponds to LO [14], a functionally defined region posterior to human MT (V5) that is topographically similar to the macaque V4 [15]. Like the macaque V4 (including the dorsal and ventral divisions), LO lies immediately posterior to MT (V5) and expands and curves postero-inferiorly, to the area anterior to VP. LO showed greater activation in response to visual images of natural objects than to a wide variety of non-object control stimuli including textures, random dots, gratings, and highly scrambled images of objects. Activation in LO did not appear to be affected by either the object's category or its level of familiarity to the subjects [15]. Hence, LO acts as an intermediate processing stage between the primary visual cortex and the higher-order "cognitive" object-recognition stages. It has been speculated that broad population coding of either object prototypes or object components underlies LO responses [15]. Recent human imaging studies suggest that subsequent, more cognitive stages involved in object identification activate more anterior and ventral areas.

The cross-modal activation revealed in the present study is in accordance with that found in previous studies [6,16]. Amedi et al. [6] first demonstrated consistent somatosensory activation in the occipito-temporal region during 3D object naming. They concluded that cortical neurons in the occipito-temporal region in humans may function as a multimodal object-selective network [6]. The activation in the occipito-temporal region reflects stored object-related visual information that can be accessed via cues from somatosensory modalities, and possibly from other modalities as well [6]. This argument suggests that direct interactions among modality-specific sensory pathways underlie the multimodal representation of objects [6]. According to this view, the bimodal activation occurred in the visual cortex rather than the somatosensory areas because object recognition relies primarily on vision. Stoesz et al. [16] reported that 2D macrospatial form perception, compared with microspatial form perception, preferentially activated the lateral occipital complex, a part of the ventral visual pathway active during visual form perception. These authors concluded that the LO is cross-modally activated during form perception.

**Figure 4:**
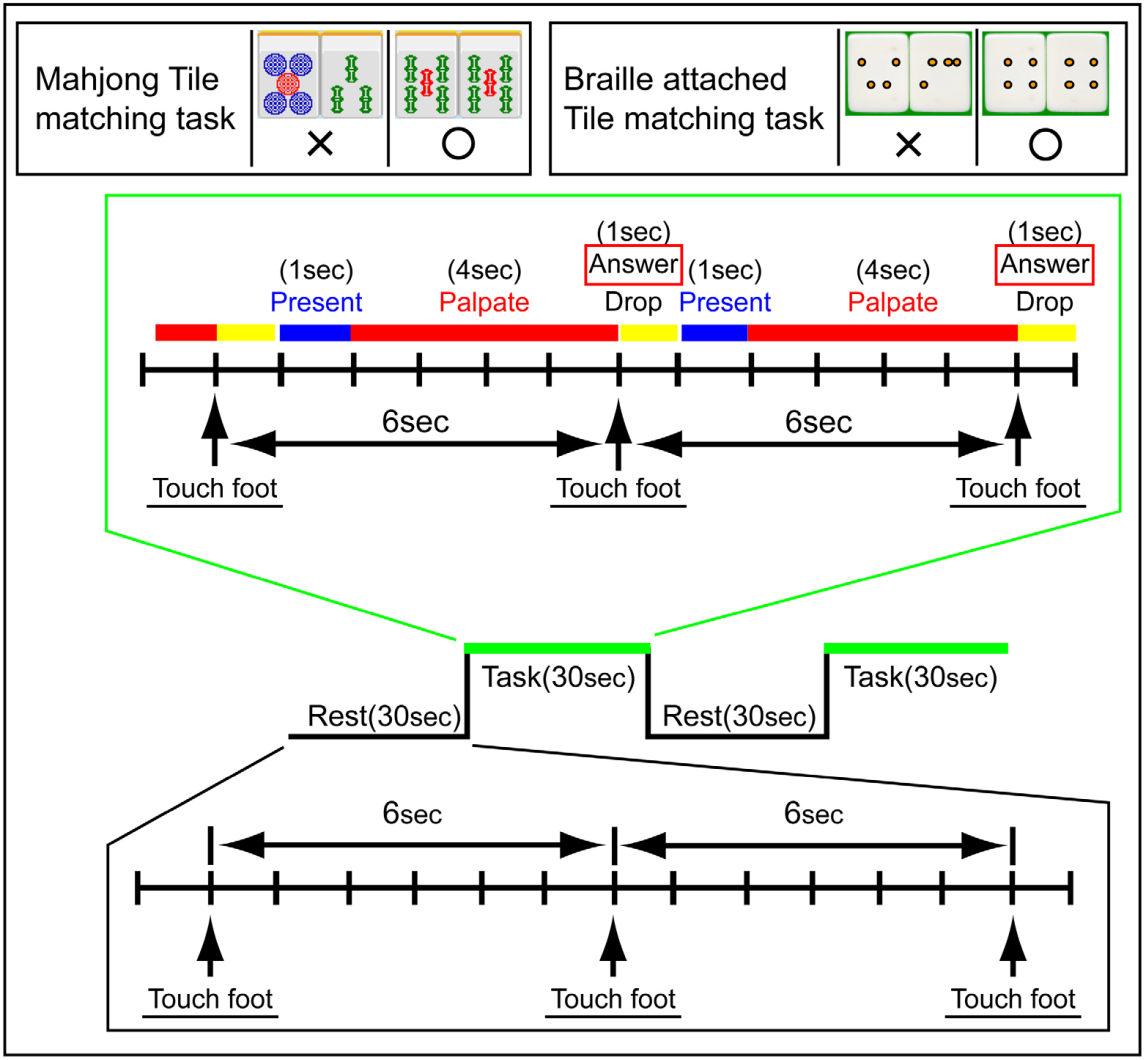
Experimental design (after [10]). Mah-Jong or Braille tile patterns were presented manually. During tactile matching, the subjects closed their eyes and a block of two joined tiles was placed in their right palm for them to feel with their right thumb. If the patterns were the same, the correct response was "yes", and subjects pressed a button with their left index finger. If the patterns were different, subjects signaled "no" by pressing a different button with the left middle finger.

The present study showed that tactile discrimination of Mah-Jong tiles activated V1 in well-trained subjects in addition to activation in the left LO. In the naïve subjects, the LO was activated but V1 was not activated. Both the LO and V1 of the well-trained subjects were activated by Braille tactile discrimination, to which all the participants were naïve. The expert group was naive to the Braille task as was the control group to the Mah-Jong task. Both groups performed comparably well for the different tasks they were both naive to. Unfortunately, we have not tested the control group to perform the Braille task but from previous studies [3,17] we can report that the performance of Braille task for naive subjects is comparable to the performance of the Mah-Jong experts that are nevertheless naive to the Braille task. Still there was a significant difference in the task-related activation of V1 between the two groups. Therefore a difference in difficulty is unlikely to be the cause of the V1 activation. The 2D patterns of the Mah-Jong tiles and Braille characters were distinctively different in terms of the concavity/convexity and the type of shape (continuous curvature vs. discrete dots) (Figure 4). Therefore, it was unlikely that the Braille characters were cross-modally associated with a visual representation. Hence the V1 activation in trained subjects was not caused by cross-modal paired associations [18,19] or visual imagery, but rather is due to cross-modal plastic changes produced by the effects of long-term training on one set of cards (Mah-Jong) that generalizes to an untrained set of cards (Braille).

Burton et al. [20] showed that simple vibrotactile stimulation activates both lower tier visuotopic (e.g., V1, V2, VP, and V3) and several higher tier visual areas (e.g., V4v, V8, and BA 37). Early blind participants showed the most extensive distribution of activity. Late blind participants exhibited activity in mostly similar regions, but the response magnitudes declined with the age of onset of blindness. Three sighted individuals had supra-threshold activity in V1. These results suggest that vibrotactile inputs probably activate the visual cortex through some latent pathway common to both blind and sighted subjects [21]. Burton et al. [22] also suggested that the learning effect may be important in the V1 activation in the blind. Using fMRI in late-onset but Braille naïve blind individuals, and auditory-presented phonological tasks, they found task-related activation in both lower tier and higher tier visual areas. Burton et al. [20] speculated that visual deprivation alone induces reorganization of the visual cortex, particularly in regions with already strong multisensory properties, where a competitive shift to non-visual inputs may readily follow visual deprivation [23]. In contrast, cross-modal reorganization of the lower tier visual areas, which are not cross-modally responsive in sighted people, may be recruited particularly through regularly attending to selected non-visual inputs. Such learning might be needed to strengthen the more remote connections with multisensory cortical areas [22]. Cross-modal reorganization of lower tier visual areas may thus be triggered in sighted subjects by learning skills such as Mah-Jong discrimination.

The present finding is in agreement with Amedi and colleagues' prediction about visual-tactile cross-modal plasticity [6]. Using blind subjects, their study showed that in the absence of visual input the somatosensory activity in the occipito-temporal region expands to the rest of the LO and to earlier retinotopic areas such as V2 and V1. Amedi et al. therefore speculated that cross-modal plasticity occurs between neighbouring areas.

The present study suggested that cross-modal activation of V1 resulting from long-term learning does not appear to have a critical period, because our subjects all acquired their skills after their mid-teens. This is in contrast with the cross-modal plasticity occurring due to sensory deafferentation. Previous studies on blind subjects who had different ages of the onset of blindness [2,3] revealed a critical period for the involvement of V1 during tactile discrimination. This discrepancy might be explained by differences in training. The sighted subjects learning Mah-Jong essentially had been performing a matching-to-sample task, i.e. matching tactile to visual information. On the other hand, blind subjects learn Braille in the tactile modality only, because they usually begin learning Braille after they have lost their sight. This point could be tested by examining whether there is a critical period of V1 activation in blind subjects for Mah-Jong discrimination. Our prediction would be that a blind person would show a critical period for Mah-Jong tile discrimination as well as Braille discrimination.

## Conclusion

The present study showed that long-term training modified the tactile-to-visual cross-modal responses in the primary visual cortex of sighted subjects. The training effect in the primary visual cortex generalized to new tactile-to-visual stimulation material but did not enhance the recognition performance for this new stimulation material.

## Methods

### Subjects

Eight Mah-Jong experts (all right-handed males; mean age 30.8 ± 6.9 years, mean training duration 9.1 ± 4.6 years) and twelve healthy volunteers who were naïve to Mah-Jong (six men, six women; mean age 29.8 ± 6.5 years) participated in this study. Eleven subjects in the control group were right-handed and one was left-handed based on the Edinburgh handedness inventory [24]. The left-handed subject did not show any significant differences when compared with the right handed group in terms of the task-related activation, and hence was included in the group analysis. None of the subjects had any history of neurological or psychiatric illness. The protocol was approved by the ethical committee of University of Fukui, and all subjects gave their written informed consent to participate in the study. The experts were highly skilful in naming the patterns of the Mah-Jong tiles by touch: they achieved more than 80% accuracy when identifying the names of the tiles by palpation. Identical training sessions before the fMRI experiments were conducted for both groups. The purpose of the training sessions was to familiarize the subjects with the task conditions. By interview, we confirmed that naïve subjects had no previous experience with the Mah-Jong game or any tactile training such as Braille reading, and that the experts also had no previous Braille training.

### Magnetic resonance imaging

In each imaging session, a time-course series of 46 volumes was acquired using T2*-weighted, gradient echo, echo planar imaging (EPI) sequences with a 3.0 Tesla MR imager (VP, General Electric, Milwaukee, WI, USA). The raw data were transferred to a parallel supercomputer (ORIGIN2000, SGI, Mountain View, CA, USA) to reconstruct the consecutive 2D images using an algorithm of 2D fast Fourier transformation (General Electric). Each volume consisted of 34 slices, each 3.5 mm thick with a 0.5-mm gap, to cover the entire cerebral and cerebellar cortex. The time interval between two successive acquisitions of the same image was 3000 ms, the echo time was 30 ms, and the flip angle was 90 degrees. The field of view (FOV) was 19 cm. The digital in-plane resolution was 64 × 64 pixels, with a pixel dimension of 2.97 × 2.97 mm. The magnetic shim was optimised such that a true in-plane resolution of 2.97 × 2.97 mm was realised. Tight but comfortable foam padding was placed around each subject's head to minimise head movement.

For anatomical reference, T2-weighted fast spin echo images were obtained from each subject with location variables identical to those of the EPIs. In addition, high-resolution whole-brain MR images were obtained using a conventional T2-weighted, fast spin echo sequence. A total of 112 transaxial images were obtained. The in-plane matrix size was 256 × 256, slice thickness was 1.5 mm, and pixel size was 0.859 × 0.859 mm. These imaging data were utilized for anatomical normalization.

### Shape-matching tasks

The task design was described previously by Saito et al. [10]. For tactile stimuli, we made 80 paired blocks of plastic Mah-Jong tiles (Taiyo Chemicals Co., Ltd, Wakayama, Japan, 26 × 18.5 × 11.6 mm) by gluing two circular- or stick-patterned tiles side-by-side. There were 40 blocks with two tiles with identical patterns and the remaining 40 blocks consisted of two tiles with two different patterns (Figure 1).

An fMRI session consisted of two rest and two task periods, each 30 s in duration, with the rest and task periods alternating. The subjects performed a tactile-tactile matching task with no visual input. The tactile matching task session was repeated twice. Prior to the fMRI session, the subjects were trained on the tactile discrimination tasks with the Mah-Jong tiles until their performance exceeded an accuracy level of 60%.

The subjects lay in a supine position with both hands extended. Their left hand was placed on the button box, which was connected to a microcomputer for recording responses. The subjects closed their eyes throughout the session. During the 30 s rest periods, the experimenter touched the subject's foot every 6 s to signal to the subject that they should push the buttons alternately with the left index finger and the left middle finger. During the task periods, a block was manually placed on the subject's right palm every 6 seconds (Figure 4). The blocks were placed so that the top of the patterns was toward the fingers. The subjects were required to explore the surface of the block with the right thumb for 4 s. When the experimenter touched the subject's foot, the subject responded by pushing a button with the left index finger if the paired patterns were the same, or with the middle finger if the patterns were different. Then the subject dropped the block. Each task period contained five trials of matching tasks, resulting in a total of 10 trials per session. Each session started with a rest condition alternating with a task condition. The session was repeated twice.

To determine if any training effect was material-specific, we conducted another functional MRI session with seven out of the eight Mah-Jong experts. The test materials were paired blocks of plastic Mah-Jong tiles, onto which two Braille characters had been pasted. The experimental conditions under which this session was carried out were identical to those used in the Mah-Jong session.

### Data analysis

The first 6 volumes of each fMRI session were discarded to allow for stabilisation of the magnetisation, and the remaining 40 volumes per session, a total of 80 volumes per subject, were used for analysis. The data were analysed using statistical parametric mapping (SPM2, Wellcome Department of Cognitive Neurology, London, UK) and implemented in Matlab (Mathworks, Sherborn, MA, USA) [25,26]. Following realignment, all images were coregistered to the high resolution, 3D, T2-weighted MRI. The parameters for affine and nonlinear transformation into a template of T2-weighted images already fitted to a standard stereotaxic space (MNI template) [11] were estimated with the high-resolution, 3D, T2-weighted MR images by least square means. The parameters were applied to the coregistered fMRI data. The anatomically normalized fMRI data were filtered using a Gaussian kernel of 8 mm (full width at half maximum) in the x, y, and z axes.

### Statistical analysis

Two levels of statistical analysis were conducted. First, we evaluated the individual task-related activation. Second, the summary data of each individual were incorporated into the second-level analysis using a random effects model [27]; this was used to make inferences at a population level regarding the task-related activation within each group and the differences between the groups.

### Individual analysis

The signal was scaled proportionally by setting the whole-brain mean value to 100 arbitrary units. The signal time course for each subject was modelled using a box-car function convolved with a hemodynamic response function, session effect, and high-pass filtering (128 s). The explanatory variables were centred at 0. To test hypotheses about regionally-specific condition effects, the estimates for each model parameters were compared with the linear contrasts. First, we delineated the areas that were active during the tasks compared with those active during the rest periods of the same session. The resulting set of voxel values for each contrast constituted a statistical parametric map (SPM) of the *t *statistic (SPM{*t*}). The SPM{*t*} was transformed to the unit normal distribution (SPM{*Z*}). The threshold for SPM{*Z*} was set at *Z *> 3.09 and *P *< 0.05, with a correction for multiple comparisons at the cluster level for the entire brain [28].

### Group analysis with random effect model

The weighted sum of the parameter estimates in the individual analysis constituted "contrast" images, which were used for the group analysis [27]. The contrast images obtained via individual analysis represent the normalized task-related increment of the MR signal of each subject. For the contrast images comparing Mah-Jong discrimination in expert and non-expert subjects, a one-sample t-test was performed for every voxel within the occipital cortex to obtain population inferences [29]. Group differences were evaluated by 2-sample t-tests. The resulting set of voxel values for each contrast constituted a statistical parametric map of the *t *statistic (SPM{*t*}). The SPM{*t*} was transformed to the normal distribution unit (SPM{*Z*}). The threshold for SPM{*Z*} was set at *Z *> 3.09 and *P *< 0.05 with a correction for multiple comparisons at the cluster level for the occipital cortex [28].

To determine whether any learning effect depicted by the between-group comparison was material-specific, Braille tactile discrimination by the expert group was evaluated at the local maximum foci detected by the between-group comparisons during Mah-Jong discrimination (Table 1). The Braille-related activation of the expert group was then compared with the Mah-Jong-related activation of the control group using Mann Whitney's U test.

## Authors' contributions

DNS carried out the MRI scanning, data analysis and drafted the manuscript.

TO operated the MRI scanner.

MH and YY participated in the task design.

NS participated in the task design, data analysis, and revision of the manuscript.

All authors read and approved the final manuscript.

## Figures and Tables

**Table 1:** Task related activation by tactile Mah-Jong discrimination

Cluster			MNI coordinates			*Z*-value	Location	BA
			
*P*	*voxel*	Size (mm^3^)	*X *(mm)	*Y *(mm)	*Z *(mm)			
Mah-Jong experts (within the occipital cortex)								

<0.001	1443	11544	10	-88	-22	4.01	GL	18
			-12	-84	-20	3.92	GL	18
			18	-72	-16	3.91	GL	18
			20	-66	-16	3.85	GL	18
			-52	-58	-8	3.84	GOm	19
			16	-80	2	3.57	GL	17/18

Expert > Non-expert (within the areas depicted by Mah-Jong experts contrast)								

0.006		1368	-8	-80	4	3.82	GL	17/18
			-6	-70	2	3.75	GL	17/18

All *P *values at cluster level were corrected for multiple comparisons over the search volume within the occipital cortex or the areas depicted by the Mah-Jong Experts contrast. GL, lingual gyrus; GOm, middle occipital gyrus.
